# Perioperative management of hemophilia A during coronary artery bypass grafting with patent foramen ovale repair

**DOI:** 10.1093/jscr/rjae578

**Published:** 2024-09-11

**Authors:** Yash P Vaidya, Theodore D Hagmann, Sara Shumway

**Affiliations:** Department of Cardiothoracic Surgery, University of Minnesota, Minneapolis, MN 55455, United States; Department of Cardiothoracic Surgery, University of Minnesota, Minneapolis, MN 55455, United States; Department of Cardiothoracic Surgery, University of Minnesota, Minneapolis, MN 55455, United States

**Keywords:** hemophilia, cardiopulmonary bypass, coronary artery bypass grafting

## Abstract

Factor VIII deficiency, also known as hemophilia A, is the most common inherited bleeding disorder. Deficiency of Factor VIII results in dysfunction of platelet aggregation due to decreased activation of Factor X to Xa. We present the case of a 68-year-old male with mild hemophilia A (Factor VIII activity, 16%) who underwent a three-vessel coronary artery bypass graft and patent foramen ovale repair, with no increased bleeding utilizing a recombinant Factor VIII (kogenate) preoperative bolus and continuous infusion. His postoperative course was complicated by a sternal wound dehiscence requiring washout, sternal wire removal and omental flap coverage on postoperative Day 21. However, he required no postoperative blood transfusions.

## Introduction

Hemophilia A is the most common inherited bleeding disorder affecting approximately 1 in 5000 male births. It is an x-linked recessive disorder of Factor VIII, a powerful activator of thrombin. Phenotypically, patients with hemophilia A are known to bleed spontaneously or excessively after minor trauma [1]. Patients with hemophilia A are known to have nearly twice the rate of coronary artery disease when compared to a white male population [2]. While the American College of Hematology has a generalized guideline for management of hemophilia in patients undergoing surgery, there are no specific recommendations for how to manage these patients while undergoing coronary artery bypass grafting (CABG). There have been a few case reports in the literature of hemophilia patients undergoing CABG with varying degrees of hemorrhagic complications [3, 4]. We present our experience of managing a patient with mild hemophilia A, who underwent CABG and PFO repair with no hemorrhagic complications.

## Case report

This patient is a 68-year-old male with past medical history significant for hemophilia A, coronary artery disease, patent foramen ovale (PFO), chronic obstructive pulmonary disease, obstructive sleep apnea, insulin dependent diabetes, hepatitis C, and prostate cancer scheduled to undergo a three vessel CABG with cardiopulmonary bypass. A random preoperative Factor VIII level was 18%. With a preoperative hematology consultation, it was planned that the patient would have a Factor VIII 50 U/kg bolus preoperatively, followed by an infusion for 14 days postoperatively. On the day of surgery, the patient had a preoperative Factor VIII level of 16%. He received a bolus infusion of 50 U/kg of kogenate (recombinant Factor VIII) 2 h prior to incision, followed by an infusion of 5 U/kg/h. Ten minutes prior to incision, the Factor VIII activity was increased to 152. Endoscopic vein harvest and cardiopulmonary bypass were uneventful. The patient was anticoagulated with heparin and cardiopulmonary bypass was established in the standard fashion with aortic and bicaval venous cannulation. Diastolic cardiac arrest was achieved with antegrade administration of 1200 ml cold blood cardioplegia. Right atriotomy was performed and the foramen ovale was closed primarily. The patient then underwent uncomplicated three vessel bypass grafting and cardiopulmonary bypass was discontinued. A bolus of 7.5 g aminocaproic acid was given and an infusion of 1.25 g/h was administered in accordance with our institutional protocol. Estimated blood loss was 1 l. The patient received a total of 450 ml cell saver volume, 800 ml blood products, and 2700 ml plasmalyte intraoperatively. The post-bypass Factor VIII activity was 105. He was transferred to the intensive care unit intubated in stable condition.

In accordance with the post-cardiac surgery enhanced recovery after surgery protocol, he was extubated within 6 h and weaned from inotropic and vasoactive support. Daily aspirin and prophylactic heparin dosing was started. Throughout the following week, his Factor VIII levels were high (>200) and the kogenate infusion was slowly weaned from 5 U/kg/h to 2 U/kg/h. This decrease in dose was necessitated by a high level of von Willebrand factor. The postoperative course was complicated by sternal wound dehiscence necessitating return to OR on postoperative Day 21 for wound washout, removal of sternal wires, and omental flap creation with the plastic surgery team. The patient was discharged to a transitional care unit one month postoperatively. He subsequently had a 3-day readmission for COPD exacerbation. He has undergone regular follow up visits, most recently at three years postoperatively where he has recovered well at home and is fully ambulatory.

## Discussion

We presented a case of coronary artery bypass grafting and PFO repair in a patient with hemophilia A with no hemorrhagic complications. While there have been reports of successful CABG procedures in patients with factor VIII deficiency, the degree of hemorrhagic complications has been variable [5]. To our knowledge, there has not been a published report of CABG with PFO repair, which changes cannulation strategy and adds to surgical complexity. There is also no established recommendation for a recombinant factor infusion regimen. Our regimen of a preoperative bolus of 50 U/kg preoperatively with a constant infusion of 5 U/kg/h titrated to Factor VIII activity assays for 14 days postoperatively was successful for postoperative management. In addition, we recommend a von Willebrand factor assay on the first postoperative day to further guide the expected response to recombinant factor infusion. With appropriate multidisciplinary support and monitoring, coronary artery bypass grafting with PFO repair can be safely undertaken in patients with hemophilia.

## References

[1] Salen P , Babiker HM. Hemophilia A. In: StatPearls [Internet]. Treasure Island (FL): StatPearls Publishing, 2024, 29261993

[2] Sharathkumar AA , Soucie JM, Trawinski B, et al. Prevalence and risk factors of cardiovascular disease (CVD) events among patients with haemophilia: experience of a single haemophilia treatment centre in the United States (US). Haemophilia 2011;17:597–604. https://doi.org/10.1111/j.1365-2516.2010.02463.x. 21323799

[3] Shalabi A , Kachel E, Kogan A, et al. Cardiac surgery in patients with hemophilia: is it safe? J Cardiothorac Surg 2020;15:76. https://doi.org/10.1186/s13019-020-01123-0. 32384896 PMC7206692

[4] Franchini M , Focosi D, Mannucci PM. How we manage cardiovascular disease in patients with hemophilia. Haematologica 2023;108:1748–57. https://doi.org/10.3324/haematol.2022.282407. 36700406 PMC10316236

[5] Menard CE , Zawadski D, Singal RK, et al. Coronary artery bypass surgery in a patient with haemophilia A: a case report. Haemophilia 2016;22:e76–9. https://doi.org/10.1111/hae.12864. 26635300

